# Assessing the Potential for Agroforestry in Pokhara, Nepal: An Investigation of the Decline of Agricultural Land Use Along a Rural–Urban Gradient and Land Suitability Analysis

**DOI:** 10.1002/pei3.70148

**Published:** 2026-04-13

**Authors:** Rajan Subedi, Sraddha Gurung, Anup Raj Adhikari, Abash Paudel, Sandip Poudel

**Affiliations:** ^1^ Institute of Forestry Tribhuvan University Pokhara Gandaki Nepal; ^2^ Arthur Temple College of Forestry and Agriculture Stephen F. Austin State University Nacogdoches Texas USA; ^3^ School of Forestry and Natural Resource Management, Institute of Forestry Tribhuvan University Kathmandu Bagmati Nepal

**Keywords:** agroforestry suitability, food insecurity, land conversion, rural‐urban migration, socio‐ecological sustainability, urbanization

## Abstract

An agroforestry program is often considered an ameliorative option for land utilization, agricultural production, and environmental sustainability. However, land suitability for agroforestry is not always readily known to farmers and local policy planners. Taking the case of Pokhara Metropolitan City in the mid‐hills of Nepal, this paper analyzes agricultural land conversion over the last three decades and identifies agroforestry suitability areas within the available agricultural land. Land conversion and urban expansion were examined using temporal Landsat imagery and population census data. Furthermore, agroforestry suitability analysis was performed using a geospatial multi‐criteria approach incorporating soil, climate, and topographic information. The study showes that during the last three decades, 11% of agricultural land was converted to built‐up areas, mainly in urban sites and 23% transitioned to forest cover in rural hills, indicating a decline in agricultural land use linked to rural to urban migration and associated land‐use dynamics. Population and household numbers were both were found to be positively correlated (*p* < 0.01) with the conversion of agriculture land to urban areas and negatively correlated with the conversion of agriculture land to forest between 1990 and 2021. The study estimates that more than half (54.3%) of the available agricultural land is highly suitable for promoting agroforestry whereas the rest (45.7%) is moderately suitable. These findings highlight a high potential for agroforestry in the Pokhara Metropolitan area, indicating significant opportunity for implementing diverse agroforestry practices within and around the city. Appropriate agroforestry intervension can enhance ecosystem services, mitigate environmental degradation, and support food security and urban green development as a nature‐based solution in rapidly urbanizing landscapes.

## 1 Introduction

Urbanization is a complex socio‐economic process that not only drives the conversion of rural areas into urban areas but also alters population distribution (DESAUN 2019). In the ensuing decades, urbanization in South and East Asia is likely to increase (UN Habitat 2020). Several push and pull factors have contributed to this rapid urbanization. The high occurrence of natural disasters in remote rural areas with exteme climate phenomenon, employment opportunities, and the availability of infrastructures like transportation, education, healthcare, public services, and communication are among the major drivers (Fort et al. 2018; Rimal 2011; Siddika and Sresto 2025). Agricultural land in Nepal is declining due to rural‐urban migration, which drives urban expansion in valleys and farmland abandonment in rural hills (Paudel et al. 2016; Subedi et al. 2025). Cultivated land is increasingly being converted to built‐up areas as metropolitan regions expand, and open spaces are rapidly transformed into urban infrastructuress (Raut et al. 2020; Rimal et al. 2018). This trend raises concerns about food insecurity and economic instability in developing countries. It also contributed to socio‐ecological imbalances in many rapidly growing cities, underscoring the need for sustainable land‐use practices.

In 2019, Nepal was ranked 73 out of 117 countries in terms of Global Hunger Index (NPC and WFP 2019), with less than 41% of the population having access to the recommended daily calorie intake. With the average agricultural landholding decreases from 1.1 ha in 1995/96 to 0.7 ha in 2010/11 (NPC 2018) hidden hunger is becoming more prominent (MoHP 2017). Agroforestry, as a means of diversified production, can reduce the vulnerability of farmers by providing short term benefits while ensuring long‐term forest products (Neupane et al. 2002). This particulalry important in a country where 53% small farmer holding less than 0.5 ha of agricultural land (CBS 2013; NPC 2018). Moreover, over two‐thirds of the land is hilly terrain where cultivation is often challenging (MoFSC 2014). Agroforestry can serve as a relatively less labor‐intensive option for generating substantial income from the available land (Dahal et al. 2020).

Agroforestry, simply the amalgam of agriculture and forestry, has been defined as “a collective name for land‐use systems and practices in which woody perennials are deliberately integrated with crops and/or animals on the same land‐management unit” (Leakey 1996). It is characterized by three key attributes: productivity, sustainability, and adoptability (Nair 1993). Agroforestry can enhance soil nitrogen fixation (Raj et al. 2025), improve soil aeration (Fahad et al. 2022), reduce water logging (Kumar et al. 2006) and support nutrient cycling, soil fertility and soil conservation (Fanish and Priya 2012). It also reduces wind erosion by acting as a windbreak (Jose 2009). In addition, agroforestry systems have the potential to store and accumulate carbon (Bajracharya et al. 2015; (Paudel et al. 2023) and serve as both adaption to and mitigation strategies for climate change challenges in the midhills of Nepal (Adhikari 2018). Agroforestry promotes biodiversity by providing green corridors (Kaushal et al. 2021) and offers a sustainable land management system that can address environmental challenges while enhancing farm productivity (Castle et al. 2021)). Furthermore, Paudel et al. (2021) reported that household practicing improved agroforestry in three mid‐hill district of Nepal generated an average annual income $841.60, along with 84% share in forest product use.

Following India in 2014, Nepal became the second country in the world to promulgate a national agroforestry policy in 2019 (Atreya et al. 2021; Chavan et al. 2015). The Government of Nepal (GoN) is further refining an agroforestry policy and piloting projects in cooperation with organizations like the International Union for Conservation of Nature (IUCN) and the Food and Agriculture Organization (FAO) (Ghimire et al. 2024). The Agriculture Development Strategy (2015–2035) and Forest Sector Strategy (2016–2025) have enlisted agroforestry in private land as a major prioritized program (MoFE 2018). Similarly, the goal of Forest Policy (2019) is aims to promote agroforestry on both public and private lands by enhancing capacity, expanding outreach, strengthening markets, supporting research, and facilitating to low interest loans (MoALD 2019). Despite these efforts, there remains for the considerable uncertainty regarding the identification of land suitable for agroforestry in Nepal. Therefore, mapping of potential land for agroforestry is essential for effective planning and intervention. However, such efforts at the local implementation level remain limited, largely due to a lack of accessible tools and spatial information. Although initiatives have been undertaken to promote agroforestry practices, they required stronger scientific support and planning.

Ning et al. (2023) showed the growing urbanization in Nepal characterized by the concentration of construction activities in cropland areas, leading to significant land fragmentation. As population growth continues to place pressure on land resources, the selection and implementation of optimal land use alternatives become increasinly important (Ahamed et al. 2000; Mazahreh 1998). Scientific land assessment is crucial for improved management and sustainable resource utilization (Ahmad et al. 2018a). The Food and Agriculture Organization (FAO) has developed a comprehensive framework for land suitability assessment that incorporates climatic, topographic, and edaphic factors (Ahamed et al. 2000). Advances in remote sensing and GIS, along with the developments in computational tools, have facilitated the application of such frameworks and expanded their use agroforestry mapping (Chen et al. 2015; Zomer et al. 2014). For example, Ritung et al. (2007) applied FAO guidelines using soil, climatic, and topographic data in Indonesia. Similarly, Yedage et al. (2013) conducted suitability mapping in Maharastra, India. Agroforestry suitability mapping has also been carried out in Dumka district of Jharkhand state, India, using FAO based methods integrated with poverty index (Ahmad and Goparaju 2017). Various parameters including soil properties, elevation, slope, rainfall, and wetness have been used in developing such suitability maps for the district (Ahmad et al. 2017, 2018b, 2019). Furthermore, the growing availability of open access data and cutting‐edge analytical methods continues to enhance GIS capabilities (Kumar et al. 2015), enabling land use planning and decision making.

Even though Nepal Agroforestry Policy (2019) emphasizes agroforestry implementation, there is limited and scattered evidence to guide policy refinement and practical application (Dhakal et al. 2022). This study employs geospatial analysis using high resolution climatic, soil, and topographical data, following FAO land evaluation guidelines to assess agricultural land suitability for agroforestry in the Pokhara Metropolitan area. It also examines the land use land cover changes and urban expansion trend over the past three decades, and analysis of agricultural land use transitions along the rural‐urban gradient using population and proximity to the national highway as proxies. By linking land suitability with land use dynamics, this integrated assessment highlight the opportunities and tradeoff for agroforestry promotion in urbanizing landscape, providing critical insights for suitable land management, food security and socio‐ecological resilience.

## 2 Materials and Methods

### 2.1 Study Area

**FIGURE 1:**
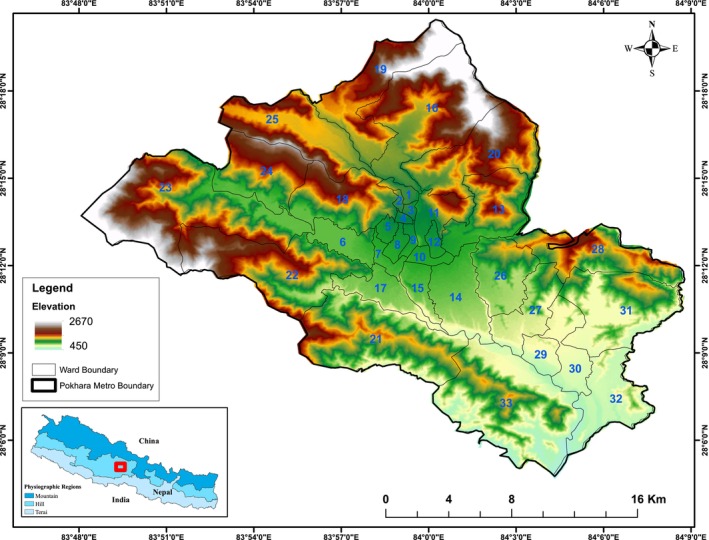
Location map of study area showing local administrative boundary (ward level) and elevation range.

Pokhara Metropolitan Municipality (28°13′ N, 83°59′ E), is the largest metropolitan with 33 wards of Nepal, coverage of 464.24 sq. km and lies in the southern aspect of the Annapurna range (Figure 1). Pokhara is surrounded by Madi and Rupa rural municipalities in the east, Annapurna rural municipality along with Parbat and Syangja districts in the west, Machhapuchhre and Madi in the north, and Syangja and Tanahun districts in the south. With an altitudinal range from 450 to 2670 m, the valley lies in subtropical climatic zone (Paudel 2020). Pokhara receives the highest amount of precipitation in the country with June, July and August as the heaviest rainfall months (Paudel 2020). It has an annual average rainfall of more than 4000 mm and an average annual temperature of around 21.5°C (Niraula and Pokhrel 2022; Paudel 2020).

Pokhara city is mostly built on flat plain land with many tourist attractions like lakes, temples, stupas, gorges, and majestic views of mountains. Being headquarters for Kaski district and Gandaki province it hosts major infrastructures such as education, health, transportation, communication, and administrative offices. Hence, urban population in Kaski, Nepal escalated to 66.08% in 2021 while it was only 13.85% in 1971 with a growth rate of 1.8% while the national rate is 0.93% (CBS 2022). The population of the city in 2021 increased to 513,504 from 413,934 in the 2011 census (CBS 2022), the increased about 24% with 2.1% annual rate. The growth is concentrated mainly in the area with good rood facility and urban services. Major highway network development and urbanization after 1950 boomed immigration in Pokhara (Poudel et al. 2025).

### 2.2 Data Used

#### 2.2.1 Satellite Imagery

The Landsat data (Table 1) downloaded from USGS Earth Explorer were used for land use land cover (LULC) classification. The selection of images data were based on image quality, especially cloud cover.

**TABLE 1:** Satellite image specifications used for land use/land cover (LULC) classification and assessment of land conversion and agroforestry suitability.

Year	Landsat sensor	Acquisition date	Path/row	Resolution	Bands used
1990	4–5 TM C1	April 16, 1990	142/40	30 m	1, 2, 3, 4, 5, 6, and 7
2000	7 ETM+ C1	December 15, 2000	142/40	30 m	1, 2, 3, 4, and 5
2010	4–5 TM C1	December 3, 2010	142/40	30 m	1, 2, 3, 4, 5, 6, and 7
2021	OLI/TIRS C1	October 14, 2021	142/40	30 m	1, 2, 3, 4, 5, 6, and 7

#### 2.2.2 Population

Population and household data for each of the 33 wards were collated from the national census of 1991, 2001, 2011, and 2021 collected from the Central Bureau of Statistics Nepal. Population trends were analyzed to understand migration patterns of different local administrative units (wards) and their connection with land conversion within the Metropolitan city.

#### 2.2.3 Road Facility

Road network map was extracted from the Open Street map (https://www.openstreetmap.org/). The national highway was used to analyze the urbanization pattern and agroforestry suitability proximate to the road network.

#### 2.2.4 Soil, Climate, and Topographical Data

The spatial layers required for agroforestry suitability analysis like nitrogen, potassium and phosphorus along with soil pH and soil organic carbon content were retrieved from Kaski Soil Fertility Assessment, 2017 in agricultural land by Regional Soil Testing Laboratory in which soil pH was determined by 1:1 Extraction Method (McLean 1982), organic matter by Modified Walkley and Black Method (Walkley and Black 1934); total nitrogen by Kjeldahl Method (Bremner and Mulvaney 1982); available phosphorus by Modified Olsen's‐Bicarbonate Method (Olsen and Sommers 1983); and available potassium by Flame Photometer Method (Thomas 1982). Uniformly spatial distribution of point data with GPS coordinate was used to prepare soil maps using Inverse Distance Weighting (IDW) for interpolation (Setianto and Triandini 2013).

The satellite image Landsat OLI data accessed of October 14, 2021, was used to generate Normalized Difference Vegetation index (NDVI) and wetness map. Similarly, ALOS PALSAR DEM of 12.5 m resolution was used for the preparation of slope and elevation map of the study area, which was downloaded from NASA Earth data website (https://asf.alaska.edu/data‐sets/sar‐data‐sets/alos‐palsar/). Mean annual precipitation map was derived based on the data from three climatic stations (Lumle, Pokhara airport, and Begnas) representing the study area managed by Department of Hydrology and Meteorology (DHM), Nepal.

### 2.3 Preparation of LULC Maps

The classification process and analysis of the different LULC classes were done using Landsat satellite image covering the Landsat 4–5 TM acquired on 16 April 1990 and 3 December 2010, Landsat satellite image covering the Landsat 7 ETM+ acquired on 15 December 2000, as well as a Landsat satellite image covering the Landsat 8 OLI/TIRS acquired on 14 October 2021. Training data sets for the classification were prepared with the help of ArcGIS 10.2.2. A simple random sampling of datasets from different classes, as observed during visualization, led to the preparation of training data. A number of training samples, identically distributed, representing each of the target classes equally were prepared. In total, more than 600 training samples were collected. The data were then saved in a shapefile format.

The R software was used for classification. The packages like sp., sf, raster and random Forest were applied. The data required for the classification, images, shape file of training datasets, and shapefile of study area were imported at first. The data were reprojected in order to match the projection. The image layers were stacked and masked before plotting the study area for visualization. Since the shapefile of training data contains the class name stored as string, it is then converted into factor data using *as.factor* function and were sorted alphabetically and numbered consecutively. After that, a data frame was created extracting input feature values and class values of every pixel from training polygons. Then a column was added to our data frame in order to match the ID of data frame and class from training datasets before deleting the ID column from the data frame. The data frame was then saved into our working directory as a.*rda* file. Using the random Forest package, a model was then prepared using *tuneRF* function after bootstrapping the sample from minority class. The classification was proceeded using *predict* function and were plotted to obtain land use land cover maps.

The Random Forest Classifier (RFC) is an ensemble classification algorithm that constructs a set of decision trees to make a prediction (Breiman 2001). Each tree is created using a randomly selected subset of training samples and variables (Nvar). By growing the forest up to a user‐defined number of trees (Ntree), the RFC creates a set of trees with high variance but low bias. RF is now one of the most extensively used methods for classifying land cover using remote sensing data (Jin et al. 2018; Maxwell et al. 2019). The reasons for RF's popularity over the last two decades are: good handling of outliers and noisy datasets (Mahdianpari et al. 2017); good performance with high dimensional and multi‐source datasets (Xia et al. 2017); higher accuracy in many applications than other popular classifiers such as SVM, kNN, or MLC (Abdel‐Rahman et al. 2014), and increasing processing speed by selecting important variables (Van Beijma et al. 2014). Each RFC run was performed using default values of Nvar (the square root of the number of predictor variables = 16 = 4) and Ntree (250) using “randomForest” package in R program. Finally, the areas of each land use were calculated based on pixels count and the count was converted into sq.km. by multiplying with the spatial resolution of the data and frequency of each land category.

For the accuracy assessment, a total of 581 points from different reference data sets were created and randomly placed in the classified image of the study area. The accuracy assessment cell array reference column was filled with each reference point's best guess. The on‐field survey of the study area generated a number of GPS points for the year 2021, which were used as ground truth data to calculate classification accuracy. Similarly for the year 2000 and 2010, reference points were collected using high‐resolution Google Earth images and while for the year 1990, topographic map (250,000 scale) published in 1988 by department of survey, Government of Nepal was used. Overall accuracy evaluation based on the agreement level suggested by Landis and Koch (1977).

### 2.4 Analysis of Land Use Transition and Urbanization

Land use transition between 1990 and 2021 was analyzed; the urbanization trend was plotted based on the urban area, classified for the years 1990, 2000, 2010, and 2021. An examination of changes was conducted for those patterns of conversion between 1990 and 2021. The land conversion pattern was analyzed for different wards unit of the metropolitan area and distance from the national highway using the arc GIS tool.

### 2.5 Agroforestry Suitability Mapping

#### 2.5.1 Conceptual Framework

**FIGURE 2:**
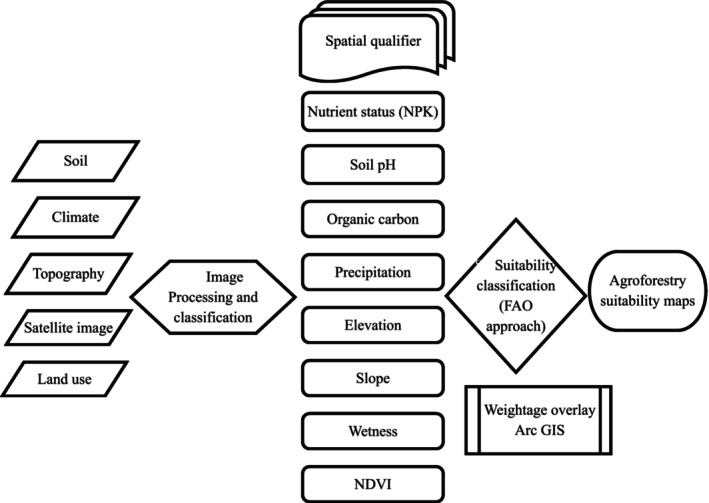
Conceptual framework adopted for agroforestry land suitability analysis on classified agricultural land.

In this study, the agroforestry suitability map was prepared based on the FAO approach (FAO 1976). The required spatial attributes were prepared from the soil, climate, topography, Landsat imagery, and land use data using Arc GIS as mentioned in Figure 2. Each layer of the thematic groups was further classified into high (S1), moderate (S2), and marginally suitable (S3) categories of sustainability, and then the final map was prepared based on the weightage overlay method as per the Table 2.

**TABLE 2:** Description of land suitability map classification scheme used for the study.

Order	Class	Description
Suitability categories	Highly suitable (S1)	Land having no significant limitations toward application of a given use, or only minor limitations that will not significantly reduce productivity and benefits and will not raise inputs above an acceptable level
	Moderately suitable (S2)	Land having limitations which in aggregate are moderately severe for sustained application of a given use, the limitations will reduce productivity or benefits and increase required inputs to the extent that the overall advantage to be gained from the use
	Marginally suitable (S3)	Land having limitations which in aggregate are severe for sustained application of a given use and will so reduce productivity and benefits, or increase required inputs, that this expenditure will be only marginally justified

#### 2.5.2 Multiple Criteria for Suitability Analysis

In the study the thematic layers under themes: soil, land, topographic, and climate were integrated together using GIS domain. Given the weight of thematic layers in decimal, weighted sum was used to conduct suitability mapping of the site. The thematic layers were integrated based on their weightage and the classification of suitability namely: Highly Suitable (S1), Moderately Suitable (S2) and Marginally Suitable (S3) which are described in given table. ArcGIS version 10.2.2 was used for spatial analysis.

##### 2.5.2.1 Classification Based Essential Nutrients

The major soil nutrients: total nitrogen, available phosphorus and available potassium were integrated together giving equal weights whose total sum equal to 100% as they are equally responsible for the metabolic growth of the plant. A similar type of mapping was done by Ahmad et al. (2019, 2018b) in Lohardaga and Ranchi of Jharkhand State, India respectively. The weights and ranks for nutrients were assigned as given in Table 3.

**TABLE 3:** Weights, ranks, and suitability classes of nutrients considered in the study.

Nutrient layers	Weight (%)	Value (kg/ha)	Ranks	Suitability
Nitrogen	33.33	> 560	3	High (S1)
		560–280	2	Moderate (S2)
		< 280	1	Marginal (S3)
Phosphorus	33.33	> 25	3	High (S1)
		25–10	2	Moderate (S2)
		< 10	1	Marginal (S3)
Potassium	33.33	> 280	3	High (S1)
		280–108	2	Moderate (S2)
		< 108	1	Marginal (S3)

##### 2.5.2.2 Integrated Agroforestry Suitability Mapping Criteria

The land qualifiers used for the suitability mapping were used as suggested by FAO which has been adopted in several research studies on agroforestry suitability mapping in India. In this study the land qualifiers: Sufficiency of Energy, Sufficiency of Water, Sufficiency of Nutrient, and Erosion degree/ease of water control each are given 25% which totals 100%. A similar type of weightage was used in studies by Ahmad et al. (2018b), and Ahmad et al. (2019) in several districts of Jharkhand State. The agroforestry suitability mapping was carried out by integrating all the land characteristics as per its relative importance‐based weight factors as shown in Table 4. The suitability of the mapping was done as per FAO classification (FAO 1976).

**TABLE 4:** Land requirement and suitability classification as per FAO guidelines for agroforestry purposes.

Land qualifies	Land characteristics	Weight (%)	Value	Ranks	Suitability
Sufficiency of energy	Elevation (m)	25	< 800 m	3	High (S1)
			800–1500 m	2	Moderate (S2)
			> 1500 m	1	Marginal (S3)
Sufficiency of water	Precipitation (mm)	12.5	> 1075	3	High (S1)
			1050–1075	2	Moderate (S2)
			< 1050	1	Marginal (S3)
	Wetness	12.5	> 0	3	High (S1)
			−0.2 to 0	2	Moderate (S2)
			< −0.2	1	Marginal (S3)
Sufficiency of nutrients	Nutrient status	8.33	High	3	High (S1)
			Medium	2	Moderate (S2)
			Marginal	1	Marginal (S3)
	Organic carbon (%)	8.33	> 0.75%	3	High (S1)
			0.75%–0.5%	2	Moderate (S2)
			< 0.5%	1	Marginal (S3)
	Soil pH	8.33	6.6–7.3	3	High (S1)
			7.4–8.4, 6.5–5.6	2	Moderate (S2)
			< 5.5	1	Marginal (S3)
Erosion degree/ease of water control	Vegetation vigorous/NDVI	12.5	> 0.3	3	High (S1)
			0.3–0.2	2	Moderate (S2)
			< 0.2	1	Marginal (S3)
	Slope angle (degree)	12.5	< 15°	3	High (S1)
			15°–35°	2	Moderate (S2)
			> 35°	1	Marginal (S3)
Ease of cultivation	Stones/rock/dams/ice and snow	—	Rock/dams/river water/ice and snow/permanent frost area		Not suitable

#### 2.5.3 Validation of Agroforestry Suitability Map

The agroforestry suitability map was validated using 46 independent, geographically referenced spatially distributed ground observation points collected in 2021, representing existing agroforestry practices within Pokhara Metropolitan City. The validation points were stratified across wards, elevation gradients, and land‐use categories to ensure adequate spatial coverage. Spatial overlay analysis was performed to assess the correspondence between observed agroforestry practices and mapped suitability classes. The points were also cross‐checked with 10‐m Sentinel‐2 NDVI data from 2020 (European Space Agency, accessed via Google Earth Engine) to ensure vegetation.

## 3 Results

### 3.1 Land Use Land Cover Change

**FIGURE 3:**
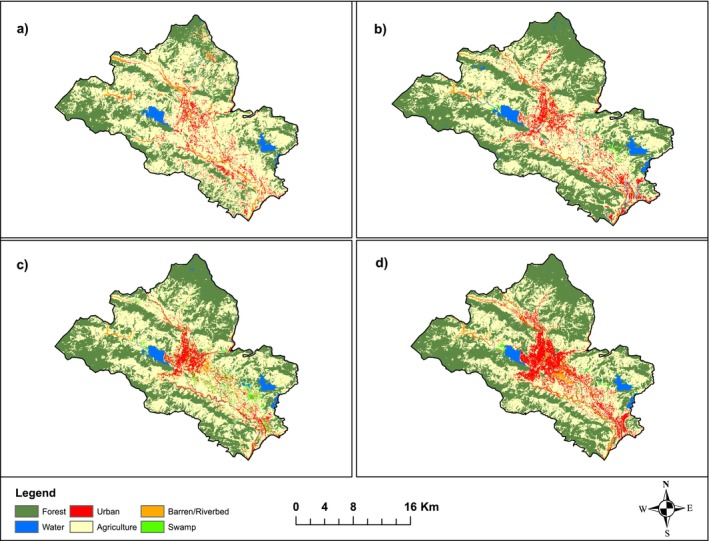
Land use and land cover (LULC) maps for (a) 1990, (b) 2000, (c) 2010, and (d) 2021, prepared for the study using Landsat imagery.

The LULC maps of Pokhara metropolitan municipality for 1990, 2000, 2010, and 2021 are shown in Figure 3; each map is classified into six land use types. The LULC map a, b, c, and d in Figure 3 belonged to 1990, 2000, 2010, and 2021, respectively. Forest covers 21,086.5 ha area which is the largest land cover type while swamp covered the lowest area in 2021. Since 1990, all types of land cover have increased at various rates except agriculture, which has decreased by 29.3%. Swamp area has increased by two‐fold, urban area by 82.7%, barren/river land by 28.3% and forest by 37%. The extent of water has stayed more or less the same since 1990 which is similar to the findings of Raut et al. (2020).

The confusion matrices for all classification years are presented in Tables S1–S4. The accuracy of classified LULC map varied between 81.18% (1990) and 87.26% (2021), with Kappa Values increasing from substantial (0.745) to almost perfect (0.84) (Table 5), reflecting improvements in reference accuracy (GPS positioning in 2021 versus topographic maps in 1990). But areas of consistent discrepancy appear: “Barren Land” had low producer accuracy (50%, 57.89% in 2021), with persistent misassignment to Agricultural or Built‐up areas (e.g., 6 in 2010, 5 in 2021), possibly because of similarities in the spectrum of mid‐hill areas with very scattered vegetation. ‘Built‐up Land’ showed variable accuracy (54.55%, 81.82% in 2021), with persistent assignment to Barren areas (3–5 for all years) because Landsat cannot distinguish very well between expanded built areas and degraded land. Forest and Swamp classes performed well producer's > 85%, user's > 84%, confirming that conversions to forest‐23% agricultural loss‐are valid. However, misclassifications of agricultural Land with forest (3–5 points) could inflate estimates of urban conversion (11%). These biases suggest that the land conversion rates presented in this study may be overestimating agricultural‐to‐urban changes by 5%–10% in marginal areas and thus overestimating claims of suitability for agroforestry at 54.3% highly suitable without finer‐resolution validation.

**TABLE 5:** Accuracy evaluation results of the land use and land cover (LULC) maps developed for the study area.

Year	Validation points	Overall accuracy (%)	Kappa coefficient	Agreement level (Landis and Koch 1977)
1990	85	81.18	0.745	Substantial
2000	154	81.82	0.80	Substantial
2010	167	85.63	0.81	Perfect
2021	175	87.26	0.84	Perfect

### 3.2 Urban Expansion, Decline in Active Cropland, and Population Dynamics

**FIGURE 4:**
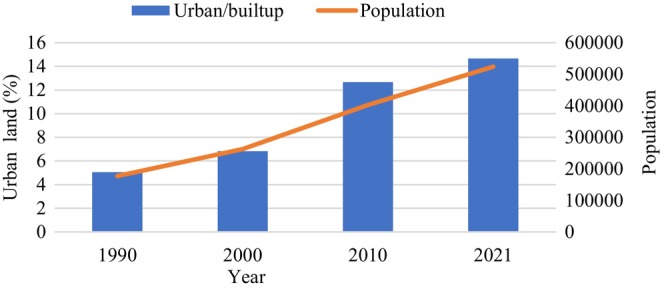
Trends in population growth and urban (built‐up) area expansion in Pokhara Metropolitan City (1990–2021), based on national census data (1991–2021) and Landsat‐derived built‐up area classification from this study.

**FIGURE 5:**
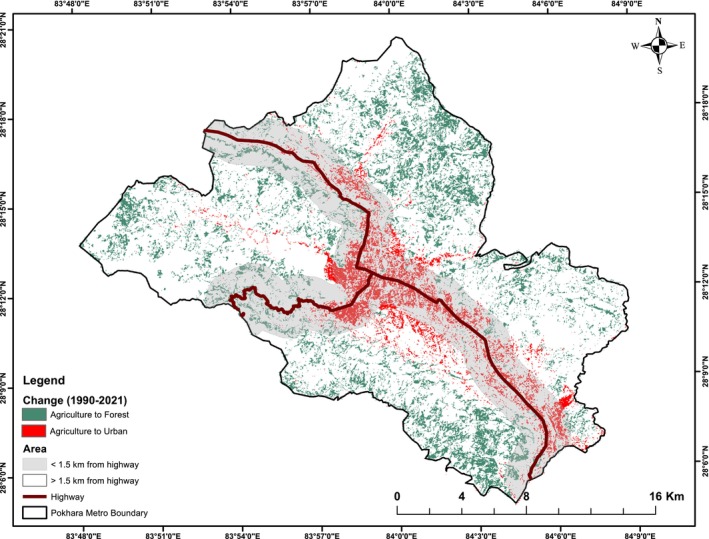
Land‐use transitions from agricultural land to forest and urban areas during 1990–2021, derived from classified land use land cover data generated in this study.

Urban areas observed rising gradually, but a higher rate of increment was found after 2000 (Figure 4). Agricultural land in Pokhara metropolitan decreased while forest areas increased between 1990 and 2021 as shown in Figure 5. The similar stagnant pattern between these two land uses between 2000 and 2010 and other patterns show increased forest area sourced from agricultural land. Similarly, the urban area gradually increased till 2010, and an abrupt rise can be noticed thereafter.

Agricultural land in 2021 covered 12009.87 ha which is 26.3% of total area while it was 28172.28 ha, that is, 58.78% of total in 1990. Since 1990, 6250.96 and 2987.98 ha agricultural land converted to forest and urban areas, respectively. Increased urbanization in this region is primarily sourced from agricultural land (Rimal et al. 2018). Among 33 wards, 11 wards, especially those at the vicinity of the highway, underwent more than 50% of agricultural land in 1990 converted to urban by 2021. Although 10 wards, especially those in rural/peri rural areas, underwent more than 30% of agricultural land to forest.

It was seen that the land conversion pattern associated with proximity to the road; the conversion of agriculture to the urban or built‐up area is higher along the highway, whereas the conversion of agriculture to forest is higher farther away from the highway.

Kendall's tau correlation coefficient was used to test the correlation significance in between agricultural land conversion with population and household change over a year at ward level (Table 6). Agricultural land converted to forest was negatively correlated to population and household increment. Whilst agricultural land converted to urban area was positively correlated with population and household change and significant at the 0.01 level.

**TABLE 6:** Correlation analysis of agricultural land conversion with population and household numbers.

Agricultural land conversion/population change	Population (1991–2020)		Household (1991–2021)	
	Coeff	Sig.	Coeff	Sig.
Agricultural land converted to forest in between (1990–2021)	−0.279	0.01*	−0.347	0.0**
Agricultural land converted to urban in between (1990–2021)	0.604	0.00**	0.569	0.00**

*Note:* The table shows correlation of agriculture land conversion with population and household.

*p < 0.05. **p < 0.01.

### 3.3 Spatial Determinants of Agroforestry Suitability

**FIGURE 6:**
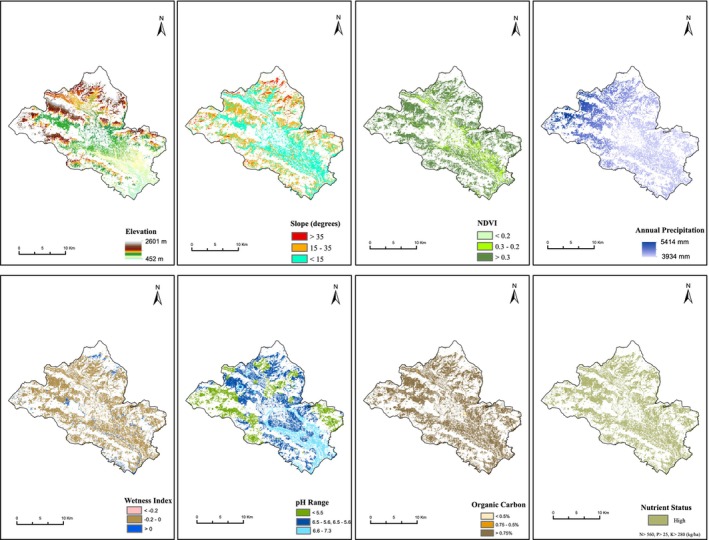
Spatial raster layers of elevation, precipitation, slope, soil pH, wetness index, NDVI, organic carbon, and cultivable land availability were prepared for the agroforestry suitability assessment.

**FIGURE 7:**
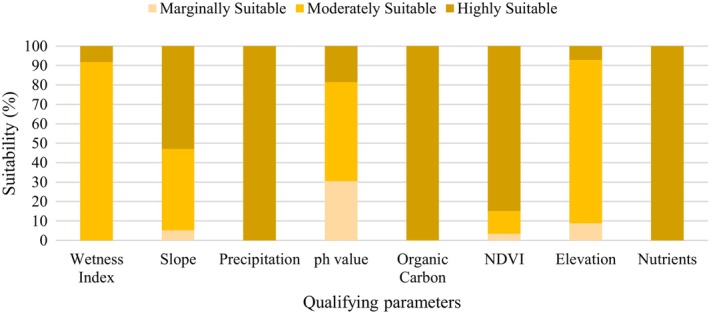
Land suitability for agroforestry (% of total active agricultural land in 2021, derived from land classification in this study) in Pokhara Metropolitan City, based on multiple suitability parameters.

The suitability categories of the applied criteria (Figure 6) were ranked based on agroforestry suitability requirements (Table 4). As a result, the total land areas of different spatial attributes layers used for the agroforestry suitability were categorized and presented in Figure 7.

Regarding the soil parameters, the nutrient status layer prepared allotting total nitrogen, available potassium, and available phosphorus with equal weightage completely support the results of high suitability to agroforestry. Similar is the condition for organic carbon. Although based on pH, less than 30% of agricultural land classified as highly suitable and around 40% moderately suitable. For the land parameters like NDVI calculated using Landsat OLI showed 84.87% high suitability, and wetness index showed 91% moderate suitability. Similarly, for the climatic parameters, Pokhara receives the most annual rainfall in Nepal. Mean annual precipitation parameter supports for high suitability.

In terms of topographical parameters, elevation of Pokhara agricultural land ranged from 452 to 2601 m, of which 84% showed moderate suitability. Similarly, only 52.9% of agricultural land had slope value less than 5°, 41% had slope value 5°–15°, and the remaining 7% had slope value 15°–35°.

The thematic values of spatial layers like soil, land, climate, and topographic parameters were assessed in highly, moderate, and marginally suitable layers (Figure 7). The analysis showed the nutrient content, organic carbon, and precipitation of the study area fall mostly into the highly suitability range. However, values of pH, wetness index, slope, and elevation limit somehow provide limitation for suitability. The present elevation‐based classification partially restricts suitability in highland areas; modifying the elevation criteria would likely increase the estimated suitable area for agroforestry in mountainous regions. Considering these factors, the current estimates reflect a conservative minimum scenario of suitable areas for agroforestry in synchronized agricultural land.

### 3.4 Agroforestry Suitability

**FIGURE 8:**
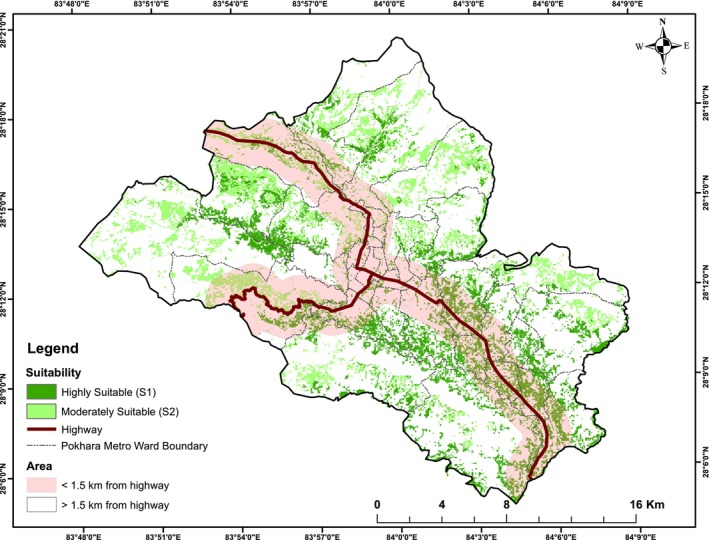
Integrated agroforestry suitability map showing highly suitable (S1) and moderately suitable (S2) classes on existing agricultural land, overlaid with local administrative boundaries (Wards 1–33) and areas proximal to roads in Pokhara Metropolitan City.

**FIGURE 9:**
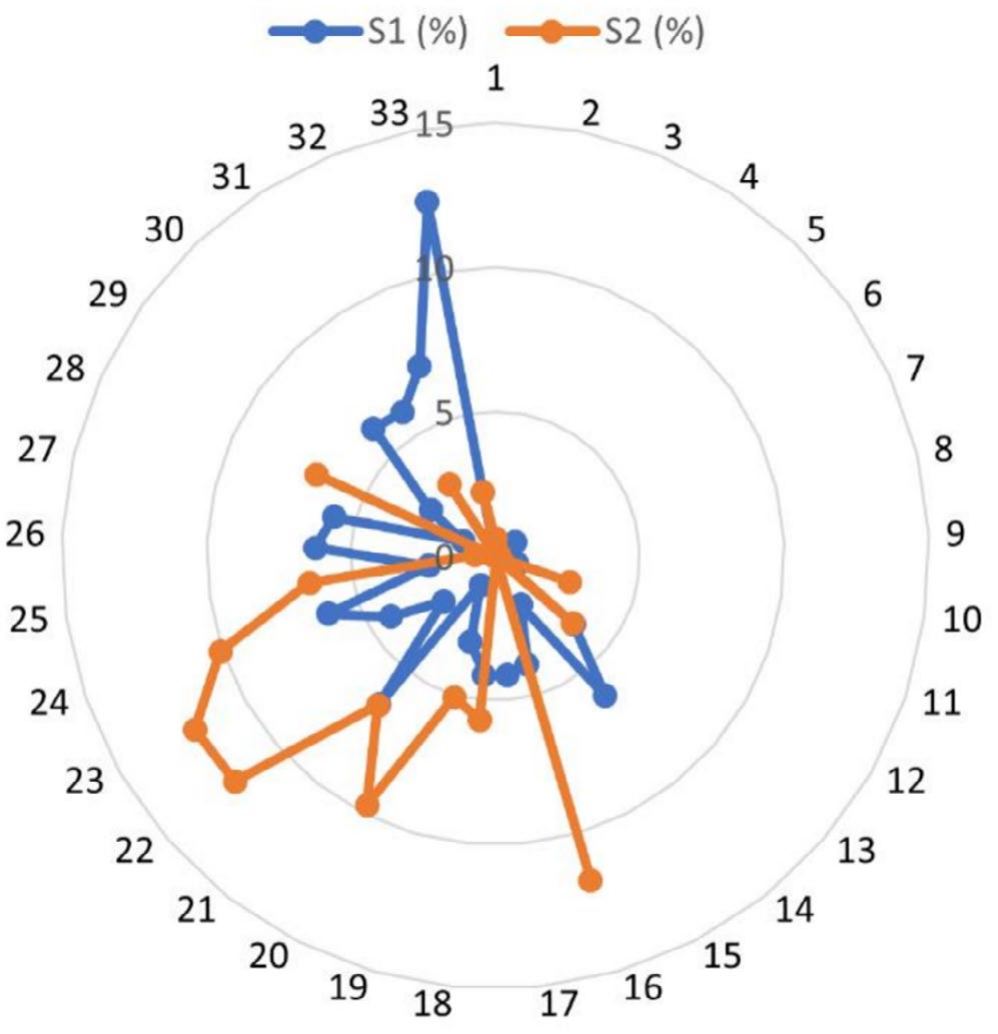
Distribution of total suitable agroforestry land across 33 administrative wards of Pokhara Metropolitan City where S1 represents highly suitable land, whereas S2 represents moderately suitable land.

In Pokhara Metropolitan, approximately 12,000 ha of agricultural land equivalent to 26.3% of the total area were identified as suitable for promoting agroforestry. Of this suitable area, 54.3% of the agricultural land was classified as highly suitable, whereas the remaining 45.7% (i.e., 5489.10 ha) was moderately suitable. The distribution of suitable agroforestry land varied across administrative wards (Figure 8). Ward No. 33 had the highest share of highly suitable land (12.49%), whereas Ward No. 2 had no land under this category. Similarly, Ward No. 23 accounted for the largest proportion of moderately suitable land, whereas Wards 1–10 and Ward 12 had negligible shares. Alongside Ward 33, Wards 14, 21, 24, 26, 27, 30, 31, and 32 each contributed more than 5% of the total highly suitable area. In contrast, Wards 16, 18, 19–25, and 28 each accounted for more than 5% of the moderately suitable land (Figure 9).

Agroforestry suitability map of Pokhara Metropolitan Municipality was further assessed against urban and rural maps drawn using the highway as a reference. Out of 12,000 ha of agricultural land in Pokhara, 69.8% of its land falls under rural and peri‐urban areas while the remaining falls under urban and urbanizing areas. Out of a total of 6208.04 ha area converted from agriculture to forest from 1991 to 2021, 83% of conversion took place in the rural area. In the case of urbanized areas converted from agriculture, 77.4% took place in urban areas.

**FIGURE 10:**
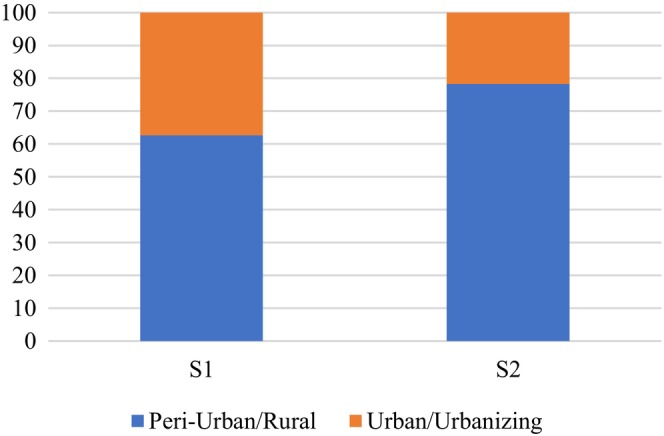
Agroforestry suitability (%) on agricultural land across rural and urban areas, with S1 representing highly suitable class and S2 representing moderately suitable class.

Rural areas in Pokhara showed high and moderate suitability almost in equal amounts. 4088.36 ha in rural areas showed high suitability which is 62.7% of total land showing high suitability in Pokhara. Similarly, 4297.44 ha moderately suitable land in rural areas comprised 78.29% of total area showing this suitability. 67.8% of urban areas, that is, 2432.41 ha high suitability and remaining shows moderate suitability (Figure 10).

**FIGURE 11:**
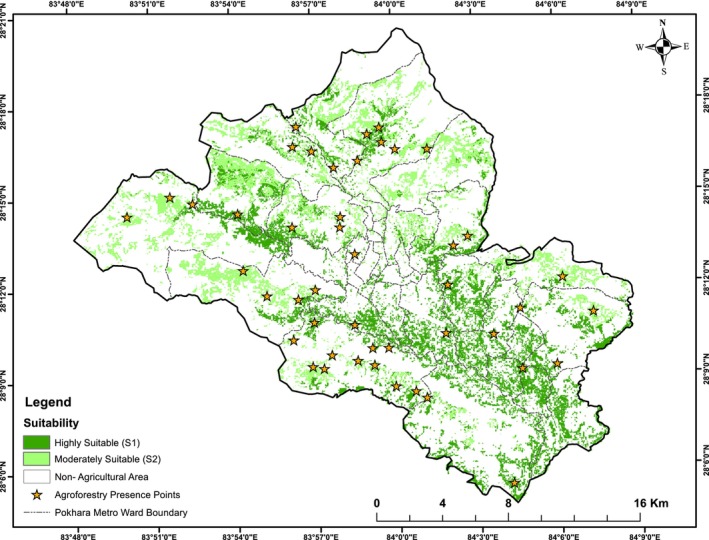
Map of observed agroforestry practice points overlaid on the agroforestry land suitability classification.

Of the 46 validation points, 44 (95.7%) were located within agricultural land classified as suitable for agroforestry. Among these, 23 points (50.0%) occurred in highly suitable (S1) zones, whereas 21 points (45.7%) were in moderately suitable (S2) zones. Only two points (4.3%) fell outside agricultural land, indicating strong spatial agreement between observed agroforestry practices and the suitability map (Figure 11).

Overall, the suitability map demonstrated an agreement of 95.7% with observed agroforestry practice locations, indicating a robust representation of agroforestry potential across agricultural lands. The two validation points located on non‐agricultural land likely reflect land‐use classification uncertainty or smallholder agroforestry practices established within forest‐classified areas.

## 4 Discussion

Our study revealed that, by 2021, forests covered the largest area in Pokhara Metropolitan City, whereas agricultural land experienced a substantial decline compared to 1990. Nearly 65% of Nepal's population depends on agriculture for livelihood (MoAC 2011), yet both Nepal and Pokhara face increasing conversion of agricultural land to non‐agricultural uses (Ojha et al. 2017; Paudel et al. 2016, 2014). Agricultural production has declined alongside a rise in food imports, reflecting a shift from Nepal being a food‐exporting nation prior to 1970 to a food‐importing country in recent decades (Pyakuryal et al. 2010). Internal migration further accelerated between 2010 and 2015 (Clewett 2015), contributing to reduced agricultural labor, lower returns from traditional farming, and heightened vulnerability to climate‐induced hazards.

Agroforestry offers a viable strategy to mitigate these challenges by diversifying returns, enhancing local livelihoods, supporting food security, and conserving biodiversity (Amatya et al. 2018). Forest regeneration under agroforestry increases carbon sequestration, improves air quality, reduces particulate matter, and enhances water retention in soils, thereby mitigating both air and water pollution. Integration of trees and vegetation on farmland also reduces soil erosion and stabilizes slopes, limiting landslide risk in the steep terrain of Pokhara's mid‐hills. Additionally, carbon credit mechanisms provide financial incentives for farmers to adopt sustainable farming systems (Awazi 2025; Raj et al. 2025). Overall, promoting agroforestry can simultaneously address food security, climate adaptation, biodiversity conservation, and natural hazard mitigation.

Urbanization in Pokhara has steadily increased over the last three decades, driven by infrastructure development and in‐migration from surrounding districts (Fort et al. 2018). Out‐migration during the 1990s, exacerbated by civil conflict and labor policy changes, contributed to agricultural land abandonment and reduced farm productivity (Jaquet et al. 2015, 2019; Raut et al. 2020). Traditional farming practices in Nepal's mid‐hills, often involving intercropping of crops with trees and livestock rearing, function effectively as agroforestry systems (Neupane et al. 2002; Paudel et al. 2016). Such systems historically met roughly 50% of household needs, whereas improved agroforestry can meet up to 84% of fodder, fuelwood, and timber demands (Paudel et al. 2021). Despite high potential suitability (> 80%) for tree integration, much agricultural land in the study area has minimal tree cover (Ahmad et al. 2021).

High suitability (S1) agricultural areas are characterized by fertile soils, sufficient precipitation, gentle slopes, and proximity to water resources, whereas moderate suitability (S2) areas exhibit more variable elevation, slope, and wetness characteristics (Ahmad et al. 2018b, 2019; Ahmad and Goparaju 2017). Elevation‐based constraints partially limit highland suitability, as 62% of sites lie above 900 m, highlighting the need for region‐specific criteria. Suitable management practices, such as agri‐horti‐silviculture in S1 areas and silvi‐pastoral systems with soil conservation interventions in S2 areas, can optimize agroforestry potential, particularly in regions with limited irrigation (Ahmad and Goparaju 2017; CBS 2013). Agroforestry in these areas also enhances slope stability, reduces sediment runoff, and mitigates flood and landslide risks in the mid‐hills (Subedi et al. 2025).

Despite the potential, geospatial analyses of site‐specific agroforestry suitability remain limited. Previous studies indicate variable suitability patterns for crops and agroforestry species across Nepal (Acharya and Yang 2015; Baniya 2008; Neupane et al. 2014). Our results suggest that Pokhara's metropolitan area is highly suitable for promoting agroforestry, offering opportunities to utilize abandoned agricultural land and meet local fodder, fuel, and timber needs (Khatri et al. 2025).

Promoting agroforestry on private lands represents a nature‐based solution for climate change mitigation, carbon sequestration, water and air pollution control, soil stabilization, and sustainable livelihoods (Dhyani et al. 2020, 2021). Integrating agroforestry under urban pressure could contribute significantly to carbon neutrality initiatives in Pokhara, especially when combined with incentive mechanisms like REDD+ (Bajracharya et al. 2015; Cedamon et al. 2018; Pandit et al. 2014).

Our land use/land cover (LULC) analysis showed that between 1990 and 2021, agricultural land declined by 29.3%, forest area increased by 37%, and urban areas expanded by 82.7%, with water extent remaining stable. Accuracy assessment of LULC maps ranged from 81.2% (1990) to 87.3% (2021), confirming reliable representation of forest gains and agricultural losses. Agricultural‐to‐urban conversion was concentrated near highways, whereas agricultural‐to‐forest transitions occurred primarily in rural and peri‐urban areas, correlating positively with population growth for urban conversion and negatively for forest conversion (Table 5). The decline in agricultural land use is linked with rural‐urban migration and associated land use dynamics. Rapid urbanization in agricultural valleys is creating pressure on land resources and productivity, while forest restoration on hill terraces and pastures presents both opportunities and tradeoff. Effective management is crucial to suport a circular economy through the sustainable management of ecosystem services, for which agroforestry can be an effecient option.

Agroforestry suitability analysis identified approximately 12,000 ha (26.3% of total area) as suitable, with 54.3% classified as highly suitable (S1) and 45.7% as moderately suitable (S2). Distribution varied across wards and between urban and rural areas, with rural lands comprising the majority of both S1 and S2 categories. Validation with 46 ground‐observed agroforestry sites showed 95.7% agreement, confirming the robustness of the suitability map. Minor discrepancies reflect land‐use classification uncertainty or smallholder practices on forest‐classified land. This indicates that most areas are favorable for agroforestry promotion and that the limitations leading to moderate suitability can be mitigated through appropriate land preparation, soil management practices, improved irrigation facilities, and water‐harvesting structures.

In summary, Pokhara Metropolitan City possesses significant potential for agroforestry adoption. Targeted promotion of agroforestry, considering site‐specific topography, soil, and climatic conditions, can provide multiple benefits including food security, carbon sequestration, biodiversity conservation, air and water quality improvement, soil erosion and landslide mitigation, and climate resilience (Akuley and Mak‐Mensah 2025; Taylor and Lovell 2021). More importantly, agroforestry implementation not only on existing croplands but also within naturally regenerated forest areas can serve as an important strategy for balancing the socio‐ecological system of a rapidly growing city. Future research on the selection of locally preferred agroforestry species and their spatial suitability is essential to ensure successful and context‐appropriate agroforestry implementation.

## 5 Conclusion

This study demonstrates that Pokhara Metropolitan City has significant potential for agroforestry adoption on agricultural lands, supported by high resolution soil, climate and topographic data. Between 1990 and 2021, agricultural land declined substantially due to urban expansion and forest regeneration linked to rural–urban migration highlighting the pressure on farmland and the opportunity to integrate trees on existing crop land for multiple benefits. Agroforestry suitability analysis identified approximately 12,000 ha (26.3% of total area) active crop land as suitable, with 54.3% classified as highly suitable and 45.7% as moderately suitable. Limitations leading to moderate suitability can be mitigated through appropriate land preparation, soil management practices, improved irrigation facilities, and water‐harvesting structures. Validation with ground‐observed agroforestry sites showed 95.7% agreement, confirming the robustness of the suitability map.

Agroforestry offers a sustainable land‐use strategy to address multiple challenges including food security, declining farm productivity, reduced motivation for conventional cropping systems, and the broader socio‐ecological sustainability of the city. Moreover, it delivers substantial environmental benefits through carbon sequestration, improvement of air and water quality, and mitigation of soil erosion and landslide risks, particularly critical in the mountainous context of Pokhara. Strategic promotion of agroforestry across rural and urbanizing areas can therefore optimize land use, enhance biodiversity, and strengthen climate resilience.

Overall, this study highlights agroforestry as a nature‐based solution with strong potential to support sustainable agriculture, environmental conservation, rural livelihoods, and climate adaptation in rapidly urbanizing mid‐hill regions of Nepal, whereas emphasizing the need for further research on site‐specific agroforestry species composition.

## Funding

The authors have nothing to report.

## Conflicts of Interest

The authors declare no conflicts of interest.

## Supporting information


**Table S1:** Class‐level accuracy assessment metrics for the 1990 land use/land cover (LULC) classification based on independent reference samples (*n* = 85).
**Table S2:** Class‐level accuracy assessment metrics for the 1990 land use/land cover (LULC) classification based on independent reference samples (*n* = 154).
**Table S3:** Class‐level accuracy assessment metrics for the 2010 land use/land cover (LULC) classification based on independent reference samples (*n* = 167).
**Table S4:** Class‐level accuracy assessment metrics for the 2021 land use/land cover (LULC) classification based on independent reference samples (*n* = 175).

## Data Availability

The datasets generated and analyzed during this study are presented in the tables and figures of the manuscript. The multi‐temporal Land Use/Land Cover (LULC) maps and agroforestry suitability layers for Pokhara Metropolitan Area used in this study are publicly available at Zenodo: https://doi.org/10.5281/zenodo.19226746.

## References

[pei370148-bib-0001] Abdel‐Rahman, E. M. , O. Mutanga , E. Adam , and R. Ismail . 2014. “Detecting Sirex Noctilio Grey‐Attacked and Lightning‐Struck Pine Trees Using Airborne Hyperspectral Data, Random Forest and Support Vector Machines Classifiers.” ISPRS Journal of Photogrammetry and Remote Sensing 88: 48–59.

[pei370148-bib-0002] Acharya, T. D. , and I. T. Yang . 2015. “Vineyard Suitability Analysis of Nepal.” International Journal of Environmental Sciences 6, no. 1: 13–19.

[pei370148-bib-0003] Adhikari, S. 2018. “Drought Impact and Adaptation Strategies in the Mid‐Hill Farming System of Western Nepal.” Environments 5, no. 9: 101.

[pei370148-bib-0004] Ahamed, T. N. , K. G. Rao , and J. Murthy . 2000. “GIS‐Based Fuzzy Membership Model for Crop‐Land Suitability Analysis.” Agricultural Systems 63, no. 2: 75–95.

[pei370148-bib-0005] Ahmad, F. , and L. Goparaju . 2017. “Geospatial Approach for Agroforestry Suitability Mapping: To Enhance Livelihood and Reduce Poverty, FAO Based Documented Procedure (Case Study of Dumka District, Jharkhand, India).” Biosciences, Biotechnology Research Asia 14, no. 2: 651–665.

[pei370148-bib-0006] Ahmad, F. , L. Goparaju , and A. Qayum . 2017. “Agroforestry Suitability Analysis Based Upon Nutrient Availability Mapping: A GIS Based Suitability Mapping.” AIMS Agriculture and Food 2, no. 2: 201–220.

[pei370148-bib-0007] Ahmad, F. , L. Goparaju , and A. Qayum . 2019. “FAO Guidelines and Geospatial Application for Agroforestry Suitability Mapping: Case Study of Ranchi, Jharkhand State of India.” Agroforestry Systems 93, no. 2: 531–544.

[pei370148-bib-0008] Ahmad, F. , M. M. Uddin , and L. Goparaju . 2018a. “Assessment of Remote Sensing and GIS Application in Identification of Land Suitability for Agroforestry: A Case Study of Samastipur, Bihar, India.” Contemporary Trends in Geoscience 7, no. 2: 214–227.

[pei370148-bib-0009] Ahmad, F. , M. M. Uddin , and L. Goparaju . 2018b. “Geospatial Application for Agroforestry Suitability Mapping Based on FAO Guideline: Case Study of Lohardaga, Jharkhand State of India.” Spatial Information Research 26, no. 5: 517–526.

[pei370148-bib-0010] Ahmad, F. , M. M. Uddin , L. Goparaju , S. K. Dhyani , B. N. Oli , and J. Rizvi . 2021. “Tree Suitability Modeling and Mapping in Nepal: A Geospatial Approach to Scaling Agroforestry.” Modeling Earth Systems and Environment 7, no. 1: 169–179.

[pei370148-bib-0011] Akuley, C. G. , and E. Mak‐Mensah . 2025. “Urban Agroforestry Practices and Their Role in Food Security and Green Space Restoration: Evidence From Kumasi, Ghana.” Agroforestry Systems 99, no. 5: 133.

[pei370148-bib-0012] Amatya, S. , E. Cedamon , and I. Nuberg . 2018. “Agroforestry Systems and Practices in Nepal.”(Revised ed.). Agriculture and Forestry University, Rampur, Nepal.

[pei370148-bib-0013] Atreya, K. , B. P. Subedi , P. L. Ghimire , S. C. Khanal , S. Charmakar , and R. Adhikari . 2021. “Agroforestry for Mountain Development: Prospects, Challenges and Ways Forward in Nepal.” Archives of Agriculture and Environmental Science 6, no. 1: 87–99.

[pei370148-bib-0014] Awazi, N. P. 2025. “The Future of Agroforestry Systems for Carbon Credits: Policy and Governance Paradigms.” In Agroforestry for Monetising Carbon Credits, 551–574. Springer.

[pei370148-bib-0015] Bajracharya, R. M. , H. L. Shrestha , R. Shakya , and B. K. Sitaula . 2015. “Agro‐Forestry Systems as a Means to Achieve Carbon Co‐Benefits in Nepal.” Journal of Forest and Livelihood 13, no. 1: 59–68.

[pei370148-bib-0016] Baniya, N. 2008. “Land Suitability Evaluation Using GIS for Vegetable Crops in Kathmandu Valley/Nepal.”

[pei370148-bib-0017] Breiman, L. 2001. “Random Forests.” Machine Learning 45, no. 1: 5–32.

[pei370148-bib-0018] Bremner, J. M. , and C. Mulvaney . 1982. “Nitrogen—Total.” Methods of Soil Analysis: Part 2 Chemical and Microbiological Properties 9: 595–624.

[pei370148-bib-0084] Castle, S. E. , D. C. Miller , P. J. Ordonez , K. Baylis , and K. Hughes . 2021. “The Impacts of Agroforestry Interventions on Agricultural Productivity, Ecosystem Services, and Human Well‐Being in Low‐ and Middle‐Income Countries: A Systematic Review.” Campbell Systematic Reviews 17, no. 2: e1167. 10.1002/cl2.1167. 37131923 PMC8356340

[pei370148-bib-0019] CBS . 2013. National Sample Census of Agriculture in Nepal. Central Bureau of Statistics, National Planning Commission, Government of Nepal (Issue December).

[pei370148-bib-0020] CBS . 2022. Nepal Census 2021: Preliminary Result, 1–96. Central Bureau of Statistics (CBS), National Planning Commission, Government of Nepal.

[pei370148-bib-0021] Cedamon, E. , I. Nuberg , B. H. Pandit , and K. K. Shrestha . 2018. “Adaptation Factors and Futures of Agroforestry Systems in Nepal.” Agroforestry Systems 92, no. 5: 1437–1453.

[pei370148-bib-0022] Chavan, S. Á. , A. Keerthika , S. Dhyani , A. Handa , R. Newaj , and K. Rajarajan . 2015. “National Agroforestry Policy in India: A Low Hanging Fruit.” Current Science 108, no. 10: 1826–1834.

[pei370148-bib-0023] Chen, Q. , D. Lu , M. Keller , et al. 2015. “Modeling and Mapping Agroforestry Aboveground Biomass in the Brazilian Amazon Using Airborne Lidar Data.” Remote Sensing 8, no. 1: 21.

[pei370148-bib-0024] Clewett, P. 2015. “Redefining Nepal: Internal Migration in a Post‐Conflict, Post‐Disaster Society.” MPI Migration Information Source. June 18, 2015. Available at, https://www.migrationpolicy.org/article/redefining‐nepal‐internal‐migration‐post‐conflict‐disaster‐society.

[pei370148-bib-0025] Dahal, G. R. , B. H. Pandit , and R. Shah . 2020. “Abandoned Agricultural Land and Its Reutilisation by Adoption of Agroforestry: A Case Study From Kaski and Parbat Districts of Nepal.” Journal of Forest and Livelihood 19, no. 1: 1–16.

[pei370148-bib-0026] DESAUN . 2019. “World Urbanization Prospects.” In World Urbanization Prospects: The 2018 Revision (ST/ESA/SER.A/420). Department of Economic and Social Affairs, Population Division, United Nations.

[pei370148-bib-0027] Dhakal, A. , T. N. Maraseni , and J. Timsina . 2022. “Assessing the Potential of Agroforestry in Nepal: Socio‐Economic and Environmental Perspectives.” In Agriculture, Natural Resources and Food Security: Lessons From Nepal, 375–394. Springer.

[pei370148-bib-0028] Dhyani, S. , A. K. Gupta , and M. Karki . 2020. Nature‐Based Solutions for Resilient Ecosystems and Societies. Springer.

[pei370148-bib-0029] Dhyani, S. , I. K. Murthy , R. Kadaverugu , R. Dasgupta , M. Kumar , and K. Adesh Gadpayle . 2021. “Agroforestry to Achieve Global Climate Adaptation and Mitigation Targets: Are South Asian Countries Sufficiently Prepared?” Forests 12, no. 3: 303.

[pei370148-bib-0085] Fahad, S. , S. B. Chavan , A. R. Chichaghare , et al. 2022. “Agroforestry Systems for Soil Health Improvement and Maintenance.” Sustainability 14, no. 22: 14877. 10.3390/su142214877.

[pei370148-bib-0030] Fanish, S. A. , and R. S. Priya . 2012. “Review on Benefits of Agro Forestry System.” International Journal of Education and Research 1:1–12.

[pei370148-bib-0031] Food and Agriculture Organization (FAO) . 1976. A Framework for Land Evaluation. Soil Bulletin, 32. Food and Agriculture Organization.

[pei370148-bib-0032] Fort, M. , B. R. Adhikari , and B. Rimal . 2018. “Pokhara (Central Nepal): A Dramatic Yet Geomorphologically Active Environment Versus a Dynamic, Rapidly Developing City.” In Urban Geomorphology, 231–258. Elsevier.

[pei370148-bib-0033] Ghimire, M. , A. Khanal , D. Bhatt , D. Dahal , and S. Giri . 2024. “Agroforestry Systems in Nepal: Enhancing Food Security and Rural Livelihoods–a Comprehensive Review.” Food and Energy Security 13, no. 1: e524.

[pei370148-bib-0034] Jaquet, S. , T. Kohler , G. Schwilch , S. Jaquet , T. Kohler , and G. Schwilch . 2019. “Labour Migration in the Middle Hills of Nepal: Consequences on Land Management Strategies.” Sustainability 11, no. 5: 1349. 10.3390/su11051349.

[pei370148-bib-0035] Jaquet, S. , G. Schwilch , F. Hartung‐Hofmann , et al. 2015. “Does Outmigration Lead to Land Degradation? Labour Shortage and Land Management in a Western Nepal Watershed.” Applied Geography 62: 157–170.

[pei370148-bib-0036] Jin, Y. , X. Liu , Y. Chen , and X. Liang . 2018. “Land‐Cover Mapping Using Random Forest Classification and Incorporating NDVI Time‐Series and Texture: A Case Study of Central Shandong.” International Journal of Remote Sensing 39, no. 23: 8703–8723.

[pei370148-bib-0037] Jose, S. 2009. “Agroforestry for Ecosystem Services and Environmental Benefits: An Overview.” Agroforestry Systems 76, no. 1: 1–10.

[pei370148-bib-0038] Kaushal, R. , D. Mandal , P. Panwar , P. Kumar , J. Tomar , and H. Mehta . 2021. “Soil and Water Conservation Benefits of Agroforestry.” In Forest Resources Resilience and Conflicts, 259–275. Elsevier.

[pei370148-bib-0039] Khatri, N. D. , S. Ghimire , and S. KC . 2025. “Meeting Forest Product Needs Through Agroforestry: Insights From Rural Mid‐Hills of Western Nepal.” Agroforestry Systems 99, no. 5: 118.

[pei370148-bib-0040] Kumar, N. , S. Yamaç , and A. Velmurugan . 2015. “Applications of Remote Sensing and GIS in Natural Resource Management.” Journal of the Andaman Science Association 20: 1–6.

[pei370148-bib-0041] Kumar, R. , S. Pandey , and A. Pandey . 2006. “Plant Roots and Carbon Sequestration.” Current Science 91, no. 7: 885–890.

[pei370148-bib-0042] Landis, J. R. , and G. G. Koch . 1977. “An Application of Hierarchical Kappa‐Type Statistics in the Assessment of Majority Agreement Among Multiple Observers.” Biometrics 33: 363–374. 884196

[pei370148-bib-0043] Leakey, R. 1996. “Definition of Agroforestry Revisited.” Agroforestry Today 8: 5.

[pei370148-bib-0044] Mahdianpari, M. , B. Salehi , F. Mohammadimanesh , and M. Motagh . 2017. “Random Forest Wetland Classification Using ALOS‐2 L‐Band, RADARSAT‐2 C‐Band, and TerraSAR‐X Imagery.” ISPRS Journal of Photogrammetry and Remote Sensing 130: 13–31.

[pei370148-bib-0045] Maxwell, A. E. , M. P. Strager , T. A. Warner , C. A. Ramezan , A. N. Morgan , and C. E. Pauley . 2019. “Large‐Area, High Spatial Resolution Land Cover Mapping Using Random Forests, GEOBIA, and NAIP Orthophotography: Findings and Recommendations.” Remote Sensing 11, no. 12: 1409.

[pei370148-bib-0046] Mazahreh, S. 1998. “Alternatives for Land Utilization in Arid to Semi‐Arid Regions in Jordan.” Unpublished Masters' Thesis, University of Jordan.

[pei370148-bib-0047] McLean, E. 1982. “Soil pH and Lime Requirement.” Methods of Soil Analysis: Part 2 Chemical and Microbiological Properties 9: 199–224.

[pei370148-bib-0048] MoAC . 2011. Priority Framework for Action Climate Change Adaptation and Disaster Risk Management in Agriculture. Ministry of Agriculture and Cooperatives, Government of Nepal.

[pei370148-bib-0049] MoALD . 2019. National Agroforestry Policy 2019. Ministry of Agri‐ culture and Livestock Development, Government of Nepal.

[pei370148-bib-0050] MoFE . 2018. Forest Policy 2018. Ministry of Environment and Foresty, Government of Nepal. Ministry of Forest and Environment.

[pei370148-bib-0051] MoFSC . 2014. Nepal National Biodiversity Strategy and Action Plan: 2014–2020, 226. Ministry of Forest and Soil Conservation, Government of Nepal.

[pei370148-bib-0052] MoHP . 2017. Nepal Demographic and Health Survey 2016. Ministry of Health and Population, Government of Nepal.

[pei370148-bib-0053] Nair, P. 1993. “State‐of‐the‐Art of Agroforestry Research and Education.” Agroforestry Systems 23, no. 2: 95–119.

[pei370148-bib-0054] Neupane, B. , C. Shriwastav , S. Shah , and K. Sah . 2014. “Land Suitability Evaluation for Cereal Crops: A Multicriteria Approach Using GIS at Parbatipur VDC, Chitwan, Nepal.” International Journal of Applied Sciences and Biotechnology 2, no. 4: 493–500.

[pei370148-bib-0055] Neupane, R. P. , K. R. Sharma , and G. B. Thapa . 2002. “Adoption of Agroforestry in the Hills of Nepal: A Logistic Regression Analysis.” Agricultural Systems 72, no. 3: 177–196.

[pei370148-bib-0056] Ning, C. , R. Subedi , and L. Hao . 2023. “Land Use/Cover Change, Fragmentation, and Driving Factors in Nepal in the Last 25 Years.” Sustainability 15, no. 8: 6957. 10.3390/su15086957.

[pei370148-bib-0087] Niraula, N. , and B. M. Pokhrel . 2022. “Changing Rainfall Pattern in Pokhara Valley, Nepal.” Geographic Base: 129–140. 10.3126/tgb.v9i1.55444.

[pei370148-bib-0057] NPC . 2018. Towards Zero Hunger in Nepal Aa Strategic Review of Food Security & Nutrition. National Planning Commission, Government of Nepal.

[pei370148-bib-0058] NPC , and WFP . 2019. The Food Security Atlas of Nepal. National Planning Commission [NPC] and World Food Program [WFP].

[pei370148-bib-0059] Ojha, H. R. , K. K. Shrestha , Y. R. Subedi , et al. 2017. “Agricultural Land Underutilisation in the Hills of Nepal: Investigating Socio‐Environmental Pathways of Change.” Journal of Rural Studies 53: 156–172.

[pei370148-bib-0060] Olsen, S. , and L. Sommers . 1983. “Phosphorus.” In Methods of Soil Analysis, edited by A. L. Page , R. H. Miller , and D. R. Keeney , 403–427. American Society of Agronomy Inc.

[pei370148-bib-0061] Pandit, B. H. , K. K. Shrestha , and S. S. Bhattarai . 2014. “Sustainable Local Livelihoods Through Enhancing Agroforestry Systems in Nepal.” Journal of Forest and Livelihood 12, no. 1: 17.

[pei370148-bib-0062] Paudel, B. , Y. Zhang , S. Li , L. Liu , X. Wu , and N. R. Khanal . 2016. “Review of Studies on Land Use and Land Cover Change in Nepal.” Journal of Mountain Science 13, no. 4: 643–660. 10.1007/s11629-015-3604-9.

[pei370148-bib-0064] Paudel, D. , K. R. Tiwari , N. Raut , et al. 2021. “Which Agroforestry Practice Is Beneficial? A Comparative Assessment of the Traditional and the Improved Agroforestry Techniques in the Midhills of Nepal.” Advances in Agriculture 2021, no. 1: 2918410.

[pei370148-bib-0063] Paudel, D. , K. R. Tiwari , N. Raut , et al. 2023. “Species Composition and Carbon Stock in Different Agroforestry Practices in the Mid‐Hills of Nepal.” Journal of Sustainable Forestry 42, no. 7: 695–711.

[pei370148-bib-0065] Paudel, K. P. , S. Tamang , and K. K. Shrestha . 2014. “Transforming Land and Livelihood: Analysis of Agricultural Land Abandonment in the Mid Hills of Nepal.” Journal of Forest and Livelihood 12, no. 1: 9.

[pei370148-bib-0066] Paudel, U. 2020. “Trends of Temperature and Rainfall in Pokhara.” Prithvi Academic Journal 3: 22–32.

[pei370148-bib-0086] Poudel, K. R. , R. B. Thapa , M. Ghimire , and R. Hamal . 2025. “Urban Growth and Urban Land Use Dynamics of Pokhara City, Nepal: Challenge to Urban Sustainability?” In Sustainability in South Asian Cities, 421–439. Springer Nature Singapore. 10.1007/978-981-97-7455-5_24.

[pei370148-bib-0067] Pyakuryal, B. , D. Roy , and Y. Thapa . 2010. “Trade Liberalization and Food Security in Nepal.” Food Policy 35, no. 1: 20–31.

[pei370148-bib-0068] Raj, A. , M. K. Jhariya , A. Banerjee , H. H. Gitari , H. K. Mina , and K. K. Mina . 2025. “Carbon Credits in Agroforestry for Net Zero Emissions: A Global Synthesis.” In Agroforestry for Monetising Carbon Credits, 113–127. Springer.

[pei370148-bib-0069] Raut, S. K. , P. Chaudhary , and L. Thapa . 2020. “Land Use/Land Cover Change Detection in Pokhara Metropolitan, Nepal Using Remote Sensing.” Journal of Geoscience and Environment Protection 8, no. 8: 25.

[pei370148-bib-0070] Rimal, B. 2011. “Urban Growth and Land Use/Land Cover Change of Pokhara Sub‐Metropolitan City, Nepal.” Journal of Theoretical and Applied Information Technology 26, no. 2: 118–129.

[pei370148-bib-0071] Rimal, B. , L. Zhang , H. Keshtkar , X. Sun , and S. Rijal . 2018. “Quantifying the Spatiotemporal Pattern of Urban Expansion and Hazard and Risk Area Identification in the Kaski District of Nepal.” Land 7, no. 1: 37.

[pei370148-bib-0072] Ritung, S. , F. Agus , and H. Hidayat . 2007. “Land Suitability Evaluation With a Case Map of Aceh Barat District.”

[pei370148-bib-0073] Setianto, A. , and T. Triandini . 2013. “Comparison of Kriging and Inverse Distance Weighted (IDW) Interpolation Methods in Lineament Extraction and Analysis.” Journal of Applied Geology 5, no. 1: 21–29.

[pei370148-bib-0074] Siddika, S. , and M. A. Sresto . 2025. “Assessing Urban Resilience of Khulna City in Response to Environmental and Socioeconomic Challenges.” DYSONA—Applied Science 6, no. 1: 134–144.

[pei370148-bib-0075] Subedi, R. , B. Jojiju , M. McBroom , et al. 2025. “Impact of Land Cover Change on Eutrophication Processes in Phewa Lake, Nepal.” Hydrology 12, no. 10: 246. 10.3390/hydrology12100246.

[pei370148-bib-0076] Taylor, J. R. , and S. T. Lovell . 2021. “Designing Multifunctional Urban Agroforestry With People in Mind.” Urban Agriculture & Regional Food Systems 6, no. 1: e20016.

[pei370148-bib-0077] Thomas, G. W. 1982. “Exchangeable Cations.” Methods of Soil Analysis: Part 2 Chemical and Microbiological Properties 9: 159–165.

[pei370148-bib-0078] UN Habitat . 2020. World Cities Report 2020: The Value of Sustainable Urbanization. United Nations Human Settlement Programme. 10.18356/c41ab67e-en.

[pei370148-bib-0079] Van Beijma, S. , A. Comber , and A. Lamb . 2014. “Random Forest Classification of Salt Marsh Vegetation Habitats Using Quad‐Polarimetric Airborne SAR, Elevation and Optical RS Data.” Remote Sensing of Environment 149: 118–129.

[pei370148-bib-0080] Walkley, A. , and I. A. Black . 1934. “An Examination of the Degtjareff Method for Determining Soil Organic Matter, and a Proposed Modification of the Chromic Acid Titration Method.” Soil Science 37, no. 1: 29–38.

[pei370148-bib-0081] Xia, J. , N. Falco , J. A. Benediktsson , P. Du , and J. Chanussot . 2017. “Hyperspectral Image Classification With Rotation Random Forest via KPCA.” IEEE Journal of Selected Topics in Applied Earth Observations and Remote Sensing 10, no. 4: 1601–1609.

[pei370148-bib-0082] Yedage, A. , R. Gavali , and A. Jarag . 2013. “Land Assessment for Horticulture (Pomegranate) Crop Using GIS and Fuzzy Decision Analysis in the Sangola Taluka of Solapur District.” International Journal of Remote Sensing and GIS 2, no. 3: 104–113.

[pei370148-bib-0083] Zomer, R. J. , A. Trabucco , R. Coe , F. Place , M. Van Noordwijk , and J. Xu . 2014. “Trees on Farms: An Update and Reanalysis of Agroforestry's Global Extent and Socio‐Ecological Characteristics.” Working Paper 179 (Ed WACISARPD WP14064. PDF). Bogor, Indonesia.

