# Supplementary material for: Assessing the Potential for Agroforestry in Pokhara, Nepal: An Investigation of the Decline of Agricultural Land Use Along a Rural–Urban Gradient and Land Suitability Analysis
Source: Plant Environ Interact. 2026 Apr 13;7(2):e70148. doi: 10.1002/pei3.70148 (PMC13071465; doi:10.1002/pei3.70148)
Supplement: Supplementary file 1 — Table S1: Class‐level accuracy assessment metrics for the 1990 land use/land cover (LULC) classification based on independent reference samples (n = 85). Table S2: Class‐level accuracy assessment metrics for the 1990 land use/land cover (LULC) classification based on independent reference samples (n = 154). Table S3: Class‐level accuracy assessment metrics for the 2010 land use/land cover (LULC) classification based on independent reference samples (n = 167). Table S4: Class‐level accuracy assessment metrics for the 2021 land use/land cover (LULC) classification based on independent reference samples (n = 175). [file PEI3-7-e70148-s001.docx]

The supplementary information presents the detailed class-level accuracy assessment results for the multi-temporal land use/land cover (LULC) classifications conducted for 1990, 2000, 2010, and 2021. Confusion matrices were generated using independent reference datasets derived from field-based GPS surveys (2021), high-resolution Google Earth imagery (2000 and 2010), and a 1:250,000 scale topographic map published in 1988 by the Department of Survey, Government of Nepal (1990). Producer’s accuracy, user’s accuracy, omission error, commission error, overall accuracy, and the Kappa coefficient were calculated for each classification year. The detail accuracy assessment results are provided in Tables S1–S4.

Table S1. Class-level accuracy assessment metrics for the 1990 land use/land cover (LULC) classification based on independent reference samples (n = 85).

| **Reference \ Classified** | **Forest** | **Agricultural Land** | **Built-up Land** | **Barren Land** | **Water Bodies** | **Swamp** | **Row Total** | **User's Accuracy (%)** |
| --- | --- | --- | --- | --- | --- | --- | --- | --- |
| **Forest** | 21 | 3 | 0 | 1 | 0 | 0 | 25 | 84 |
| **Agricultural Land** | 4 | 22 | 1 | 3 | 0 | 0 | 30 | 73.3 |
| **Built-up Land** | 0 | 2 | 6 | 3 | 0 | 0 | 11 | 54.55 |
| **Barren Land** | 0 | 0 | 0 | 7 | 1 | 0 | 8 | 87.5 |
| **Water Bodies** | 1 | 0 | 0 | 0 | 7 | 0 | 8 | 87.5 |
| **Swamp** | 0 | 0 | 0 | 0 | 0 | 3 | 3 | 100 |
| **Column Total** | 26 | 27 | 7 | 14 | 8 | 3 | 85 |  |
| **Producer's Accuracy (%)** | 80.77 | 81.48 | 85.71 | 50 | 87.5 | 100 |  | **Overall: 81.18%**  **Kappa: ~0.745** |

Table S2. Class-level accuracy assessment metrics for the 1990 land use/land cover (LULC) classification based on independent reference samples (n = 154).

| **Reference \ Classified** | **Forest** | **Agricultural Land** | **Built-up Land** | **Barren Land** | **Water Bodies** | **Swamp** | **Row Total** | **User's Accuracy (%)** |
| --- | --- | --- | --- | --- | --- | --- | --- | --- |
| Forest | 40 | 5 | 0 | 1 | 0 | 0 | 46 | 86.96 |
| Agricultural Land | 6 | 49 | 2 | 4 | 0 | 0 | 61 | 80.33 |
| Built-up Land | 0 | 2 | 11 | 3 | 0 | 0 | 16 | 68.75 |
| Barren Land | 0 | 0 | 0 | 11 | 1 | 0 | 12 | 91.67 |
| Water Bodies | 1 | 0 | 0 | 0 | 10 | 0 | 11 | 90.91 |
| Swamp | 0 | 0 | 0 | 0 | 0 | 8 | 8 | 100 |
| Column Total | 47 | 56 | 13 | 20 | 11 | 8 | 154 |  |
| Producer's Accuracy (%) | 85.1 | 87..5 | 84.61 | 55 | 90.9 | 100 |  | Overall: 81.82% Kappa: ~0.80 |

Table S3: Class-level accuracy assessment metrics for the 2010 land use/land cover (LULC) classification based on independent reference samples (n = 167).

| **Reference \ Classified** | **Forest** | **Agricultural Land** | **Built-up Land** | **Barren Land** | **Water Bodies** | **Swamp** | **Row Total** | **User's Accuracy (%)** |
| --- | --- | --- | --- | --- | --- | --- | --- | --- |
| **Forest** | 42 | 2 | 0 | 0 | 0 | 0 | 44 | 95.45 |
| **Agricultural Land** | 4 | 55 | 0 | 6 | 0 | 0 | 65 | 84.62 |
| **Built-up Land** | 0 | 2 | 18 | 2 | 0 | 0 | 22 | 81.82 |
| **Barren Land** | 0 | 3 | 0 | 11 | 1 | 0 | 15 | 73.33 |
| **Water Bodies** | 1 | 0 | 0 | 2 | 8 | 0 | 11 | 72.73 |
| **Swamp** | 0 | 0 | 0 | 1 | 0 | 9 | 10 | 90 |
| **Column Total** | 47 | 62 | 18 | 22 | 9 | 9 | 167 |  |
| **Producer's Accuracy (%)** | 89.36 | 88.71 | 100 | 50 | 88.9 | 100 |  | **Overall: 85.63% Kappa: ~0.81** |

Table S4: Class-level accuracy assessment metrics for the 2021 land use/land cover (LULC) classification based on independent reference samples (n = 175)

| **Reference \ Classified** | **Forest** | **Agricultural Land** | **Built-up Land** | **Barren Land** | **Water Bodies** | **Swamp** | **Row Total** | **User's Accuracy (%)** |
| --- | --- | --- | --- | --- | --- | --- | --- | --- |
| **Forest** | 48 | 0 | 0 | 0 | 0 | 0 | 48 | 100 |
| **Agricultural Land** | 1 | 57 | 0 | 5 | 0 | 0 | 63 | 90.48 |
| **Built-up Land** | 0 | 4 | 21 | 5 | 0 | 0 | 30 | 70 |
| **Barren Land** | 1 | 3 | 1 | 12 | 3 | 0 | 20 | 60 |
| **Water Bodies** | 0 | 0 | 0 | 0 | 8 | 0 | 8 | 100 |
| **Swamp** | 0 | 0 | 0 | 0 | 0 | 6 | 6 | 100 |
| **Column Total** | 50 | 64 | 22 | 22 | 11 | 6 | 175 |  |
| **Producer's Accuracy (%)** | 96 | 89.06 | 95.45 | 54.55 | 72.73 | 100 |  | **Overall: 87.26% Kappa: ~0.84** |
